# Editorial: Novel Strategies Targeting Obesity and Metabolic Diseases

**DOI:** 10.3389/fphys.2019.00668

**Published:** 2019-05-29

**Authors:** Xinran Ma, Dechun Feng, Yan Lu, Nuo Sun, Jiqiu Wang, Lingyan Xu

**Affiliations:** ^1^Shanghai Key Laboratory of Regulatory Biology, Institute of Biomedical Sciences and School of Life Sciences, East China Normal University, Shanghai, China; ^2^National Institute on Alcohol Abuse and Alcoholism, National Institutes of Health, Bethesda, MD, United States; ^3^Department of Endocrinology and Metabolism, Zhongshan Hospital, Fudan University, Shanghai, China; ^4^Wexner Medical Center, The Ohio State University, Columbus, OH, United States; ^5^National Key Laboratory for Medical Genomes, Department of Endocrinology and Metabolism, China National Research Center for Metabolic Diseases, Ruijin Hospital, Shanghai Jiao Tong University School of Medicine, Shanghai, China

**Keywords:** obesity, metabolic disease, pathophysiology, treatment, diagnosis

The obesity pandemic poses one of the most serious problems threatening public health with its staggering morbidity/mortality rate and its close link to multiple diseases including diabetes, cardiovascular disease, hypertension, non-alcoholic hepatic steatosis, and certain types of cancer. The imbalance of energy intake/expenditure and the genetic susceptibility of the individual both impact obesity development. With the prevalent sedentary life style in modern society, the obesity incidence soars yet efficient pharmacotherapeutic solutions targeting appetite or energy expenditure are limited and often with undesired side effects. Nowadays, it is urgent to seek better understandings of obesity to seek effective strategies that target obesity and related metabolic diseases. With the opportunity of the current Frontiers Research Topic, we focus on obesity and discuss analytic tools for obese phenotypes, potential therapeutic gene targets, as well as novel strategies against obesity and over-nutrition induced metabolic derangements, with the hope to provide a comprehensive summary of the latest metabolic studies.

Obesity features excessive lipid accumulation in adipose tissues. As the saying goes, “a handy tool makes a handyman,” it makes good sense to start this research topic with handy tools accessing the obese phenotypes. Zhi et al. present a novel adipocyte counting system, AdipoCount, which provides automatic quantification of the number and diameter of adipocytes with higher accuracy and supports manual correction compared to existing tools. It serves as a necessary step to accurately access the obese phenotype in humans and animal models for further scientific studies.

Secondly, we discuss advances in our understanding in the development of obesity and potential therapeutic targets against it. Research article from Wang et al. reveals novel functions of GPR54, a family member of G protein-coupled receptors (GPCRs), in promoting adipocyte differentiation and triglyceride accumulation by regulating lipogenic genes including PPARγ via ERK1/2 phosphorylation. GPR54 deficient mice are resistant to central obesity, insulin resistance, and inflammation under high fat diet (HFD). Thus, it would be worthwhile to develop GPR54 inhibitors as potential treatment for obesity and metabolic diseases.

Unlike GPR54, a relatively novel player in the metabolic field, the functions of AMP-Activated Protein Kinase (AMPK) are extensively studied in many metabolic organs. However, how AMPK regulates energy metabolism in adipose tissues is not fully understood. Using Adipose tissue-specific AMPK α1/α2 KO (AKO) mice, Wu et al. demonstrate that AMPKα deficiency in adipocytes causes impairment in the browning capability in inguinal fat (iWAT, mainly consists of beige adipocytes). AMPKα KO mice are cold intolerant and poised to obesity, whereas chronic AMPK activation by its allosteric activator A-769662 promotes iWAT browning and protects mice against diet-induced obesity and related metabolic dysfunction. It is well established that beige fat plays a vital role in promoting thermogenesis and energy expenditure, as well as in maintaining glucose and lipid homeostasis as metabolic sinks in both rodents and human. Thus, Wu et al's. report lends strong support to implicating AMPK agonists as potential therapeutics for obesity and metabolic diseases.

Meanwhile, in the review article from Kuang et al., spotlight is focused on another key regulator in obesity and metabolic diseases, Sirt6. As an important member of the Sirtuin family, Sirt6 impacts multiple physiological and pathological processes including aging, cancer, obesity, and energy metabolism. Sirt6 level and function decrease under aging and over nutrition, resulting in abnormal glucose and lipid metabolism. *In vivo* studies indicate that Sirt6 deficiency promotes diet-induced obesity, insulin resistance and liver steatosis, suggesting a protective role of Sirt6 activation of in obesity and diabetes.

Thirdly, in the everlasting quest for effective therapeutic leads targeting obesity and metabolic diseases, we discuss novel inspirations emerging from areas both new and old. Gut microbiota, a relatively new yet critical player in the battle against obesity, has been shown to actively contribute to the pathogenesis and intervention of obesity through their interaction with the host, a myriad of metabolites they produce, or their dynamic composition, all of which are in turn influenced by diet. In the systematic review from Zhang et al. the impacts of dietary substrates and host genotype/enterotype on microbiota composition and metabolites are discussed. These factors contribute to the maintenance of a health commensal homeostasis between gut microbiota and the host, which is vital for host metabolic fitness and obesity prevention. In addition, Madsen et al. focus on the impact of different dietary protein sources on gut microbiota. Different dietary protein sources vary in amino acid composition and other factors such as fatty acids and pollutions, which could modulate the composition of gut microbiota and thereby affect energy efficiency and obesity development. These studies emphasize the importance of gut microbiota on metabolic health, while at the same time offer dietary intervention as a promising strategy in combating obesity and metabolic diseases.

In the original study from Sun's lab, a daily supplement, branched-chain amino acid (BCAA), is highlighted. BCAA (including essential amino acids leucine, isoleucine, and valine) has been shown to be beneficial for metabolic fitness by regulating lipid and glucose homeostasis in liver and adipose tissues, as well as protein synthesis and degradation in skeleton muscle. In this regard, Liu et al. demonstrate that BCAA negatively targeting Krüppel-like factor 15, a master regulator of glucose, lipid, and amino acids metabolism, via PI3K-AKT signaling, which offer new mechanistic insights underlying BCAA's multiple functions in metabolic regulation.

On the other hand, two articles explore the possibility of finding effective therapeutic strategies from traditional Chinese medicine. Zhao et al. examine the effects of Er-Miao-Fang, a traditional Chinese Medical prescription, on HFD-induced adipose tissue dysfunction and find that its extracts reduce fatty acids and glycerol release from adipose tissue, thus block fatty acids and inflammatory molecules trafficking from adipose tissues to the liver, which in the end inhibits adipose tissue inflammation and ameliorates hepatic steatosis and insulin resistance under HFD. Likewise, Du et al. find that Astragaloside IV, the main active ingredient in the medicinal herb *Astragalus membranaceus*, suppresses adipocyte HSL activity through cAMP/Akt/PDE3B signaling, which results in reduced lipolysis and decreased circulating free fatty acid levels, thus limits hepatic lipid deposition and restrains excessive hepatic glucose production.

Last but not least, obesity and its associated metabolic derangements, i.e., chronic low-grade inflammation, insulin resistance, abnormal lipid profiles, often lead to a range of diseases and complications including diabetes, cardiovascular diseases, renal pathological changes etc., which are our next focus of discussion. For example, diabetic cardiomyopathy (DCM) is partially initiated by prolonged disturbances in energy substrates in diabetes. With ultra-high-performance liquid chromatography coupled to a quadruple time of flight mass spectrometer (UPLC/Q-TOF/MS) approach, Dong et al. reveal lipidomic biomarkers in the rat model of diabetic cardiomyopathy including 17 potential biomarkers of phosphatidylcholines, phosphatidylethanolamines, and sphingolipids. They also offer berberine administration as an effective treatment in the protection against cardiac diastolic and systolic dysfunctions partially via reversing the lipid profiles. With similar strategy, Li et al. evaluate lipid metabolites in endometriosis and indicate phosphatidylcholine (18:1/22:6), (20:1/14:1), (20:3/20:4), and phosphatidylserine (20:3/23:1) and increased phosphatidic acid (25:5/22:6) as early diagnostic biomarkers for endometriosis.

Prolonged diabetes may lead to the development of diabetic nephropathy (DN). Zhang et al. report the inhibition of (pro)renin receptor (PRR) contributes to renoprotection against DN by Angiotensin II type 1 receptor (AT1R) blockade. They thus highlight a potential therapeutics for renoprotection using combined blockade of AT1R with losartan, and PRR with a decoy peptide of prorenin.

Additionally, the study from Fan et al. links male subfertility with obesity-induced chronic inflammatory status in male genital tract, while Yang et al. show that immune status impacts insulin sensitivity in Hashimoto's Thyroiditis (HT) patients and HT mice. In HT mice, reinfusion of regulatory T cells (Tregs) from peripheral blood of normal mice improves insulin sensitivity and decreases inflammation. They also report increased resident Tregs and enhanced cytokine production in visceral adipose tissues (VAT) in HT mice, suggesting a critical role of VAT resident Tregs in HT associated insulin resistance.

In conclusion, the current research topic provides comprehensive and in depth understandings on various aspects of the researches on obesity, offering novel quantitative methods, diagnostic biomarkers, targeting molecules, and their action of mechanisms, as well as potential therapeutics toward obesity and metabolic diseases. Together, these studies would facilitate future development of effective prevention, diagnosis, and personalized therapeutic strategies to combat obesity and other metabolic diseases.

## Author Contributions

XM and LX conceived and drafted the editorial. DF, YL, NS, and JW critically revised the work and approved its version to be submitted.

### Conflict of Interest Statement

The authors declare that the research was conducted in the absence of any commercial or financial relationships that could be construed as a potential conflict of interest.

